# The Influence of Phosphoinositide Lipids in the Molecular Biology of Membrane Proteins: Recent Insights from Simulations

**DOI:** 10.1016/j.jmb.2025.168937

**Published:** 2025-01-09

**Authors:** George Hedger, Hsin-Yung Yen

**Affiliations:** 1 Department of Life Sciences, Sir Ernst Chain Building, Imperial College London, London, SW7 2AZ, UK; 2 Institute of Biological Chemistry, Academia Sinica, Taipei 115, Taiwan; 3 Department of Chemistry, University of Oxford, South Parks Road, Oxford, OX1 3QZ, UK

**Keywords:** phosphoinositide, PIP_2_, lipid-protein interaction, MD simulation, membrane protein

## Abstract

The phosphoinositide family of membrane lipids play diverse and critical roles in eukaryotic molecular biology. Much of this biological activity derives from interactions of phosphoinositide lipids with integral and peripheral membrane proteins, leading to modulation of protein structure, function, and cellular distribution. Since the discovery of phosphoinositides in the 1940s, combined molecular biology, biophysical, and structural approaches have made enormous progress in untangling this vast and diverse cellular network of interactions. More recently, *in silico* approaches such as molecular dynamics simulations have proven to be an asset in prospectively identifying, characterising, explaining the structural basis of these interactions, and in the best cases providing atomic level testable hypotheses on how such interactions control the function of a given membrane protein. This review details a number of recent seminal discoveries in phosphoinositide biology, enabled by advanced biomolecular simulation, and its integration with molecular biology, biophysical, and structural biology approaches. The results of the simulation studies agree well with experimental work, and in a number of notable cases have arrived at the key conclusion several years in advance of the experimental structures.

## Introduction

Phosphoinositides are a family of membrane lipids with far reaching influences on cellular function.^1^ The study of phosphoinositides has a rich history dating back many decades.^2–7^ This work has revealed roles in cell signalling,^8^ trafficking,^9^ cytoskeletal organisation,^10^ and regulation of ion passage across membranes.^11^ Pathogenic variance in the kinases and phosphatases which control the relative cellular abundance of phosphoinositides are associated with a number of human diseases including growth disorders, cancers, and neurological conditions.^12–16^ Underlying these cellular functions are molecular level interactions of phosphoinositides with integral and peripheral membrane proteins. Experimental methods such as X-ray crystallography have made significant early contributions to revealing the detailed architecture of membrane proteins in complex with phosphoinositides.^17^ The advent of technologies including atomic resolution cryo-EM^18^ and native mass spectrometry^19^ have accelerated that understanding.^20–22^ Such structural studies have been coupled to intricate biochemical work to show how specific phosphoinositides control the function of certain membrane proteins. Despite progress, resolving bound phosphoinositides in structures^23^ and relating this information to function and dynamics at the molecular-level using experimental techniques remains non-trivial from static structures alone. *In silico* approaches such as molecular dynamics (MD) simulations have experienced rapid technological development in the area of membranes and membrane proteins.^24,25^ This has led to a surge in simulation studies, and a number of notable contributions to uncovering new aspects of phosphoinositide biology. Many of these simulations have been performed in an integrated fashion with experiments. The simulations serve to computationally reunite experimentally determined membrane protein structures with their complex lipid bilayer environment, and then capture the atomic level dynamics and structural aspects of how the membrane protein interacts with phosphoinositides. In a number of cases, simulations have prospectively predicted phosphoinositide binding sites on integral membrane proteins several years in advance of experimental structures being determined. In the very best examples the simulations have provided a detailed mechanistic view into how the structure and dynamics of the interaction then lead to the phosphoinositide altering the function of a given membrane protein.

Within this review we examine how simulations and complementary experiments have revealed new and exciting molecular-level aspects of phosphoinositide biology. We begin with a brief survey of cell membranes, phosphoinositides, and biomolecular simulation approaches.

### Cell membranes

Cells and subcellular compartments are defined by membranes. Membranes are composed of a lipid bilayer and embedded membrane proteins. This bilayer is compositionally complex, containing hundreds of lipid species asymmetrically distributed between leaflets.^26^ Advances in structural biology of membrane proteins^18^ and in lipidomics^27,28^ have led to enormous progress in uncovering the compositional complexity and molecular level architecture of membranes. The structures of ca. 1700 unique membrane proteins have been determined (https://blanco.biomol.uci.edu/mpstruc/) alongside ca. 49,000 lipid structures (https://www.lipidmaps.org/databases/lmsd/overview). The composition of a membrane and its organisation varies substantially by membrane type, stage in the life cycle, metabolic state, signalling state, and health and disease.^29,30^ Lipids interact with membrane proteins and alter their structure, function, localisation, and oligomeric states.^31^ These interactions may take a variety of forms, including binding to specific well-defined sites on the membrane-exposed surface of a protein with complementary geometry, binding to sites deep within a protein, or more dynamic interactions with disordered patches of complementary charge or grease. They may also occur indirectly by influences on the local biophysical properties of the membrane. Lipids may even represent the native ligand for a membrane protein, as has been proposed for activation of Smoothened by cholesterol.^32,33^ Examples of regulation of membrane protein function by phosphoinositides include receptor tyrosine kinases (RTKs),^34^ inward rectifying potassium (Kir) channels,^35^ G-protein-coupled receptors (GPCRs),^21^ neurotransmitter transporters^36,37^ and syntaxins.^38^ In the case of phosphoinositides, another major type of lipid-protein interaction is the recruitment and orientation of soluble proteins, so called ‘peripheral membrane proteins’ (PMPs) to the membrane surface by direct association of the PMP with the phosphoinositide headgroup.^39,40^

### The phosphoinositide family

**Figure 1:**
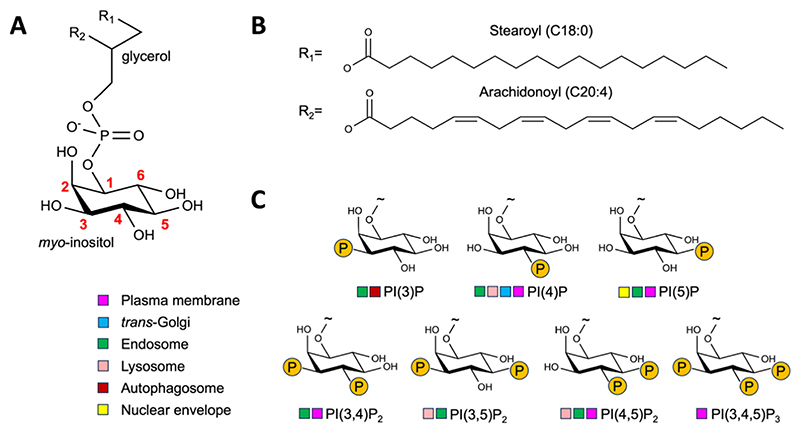
The phosphoinositide family. (A) Structure of the phosphatidylinositol headgroup depicting the chair conformation and numbering scheme of the myo-inositol moiety. (B) The predominant fatty acid tails. (C) Headgroup structures of the seven eukaryotic phosphoinositides, derived from phosphatidylinositol. The average cellular distributions are indicated. Note that this varies with cell type and myriad other variables. See Refs. 14,44 for further detailed discussion of cellular distribution.

Phosphoinositides are phosphorylated derivates of phosphatidylinositol (PI) (Figure 1). Differential decoration of the *myo*-inositol headgroup with varying numbers and positions of phosphate groups give rise to seven derivates in humans. The inositol group adopts a chair conformation with inorganic ions complexed to the negative phosphoryl groups.^41^ The acyl tails show unusual uniformity compared to other phospholipids, and are usually of the 1-stearoyl-2-arachidonoyl variety.^42^ The overall abundance of phosphoinositides is estimated at less than 1% of cellular phospholipids, with species such as PI(3,4,5)P_3_ being even rarer.^43,44^ This seeming dichotomy led to the famous adage ‘Tiny lipids with giant impact on cell regulation’.^1^ Precise measurements of abundance remain challenging, not least as it is a moving target. The relative abundance of different phosphoinositide species is under constant dynamic spatiotemporal control, and varies by cell type, signalling state, and myriad other cellular conditions. The distribution of phosphoinositide species also varies by subcellular location (Figure 1C),^14,44,45^ which confers unique biochemical properties to the host membranes. Localisation to specific membranes, as well as localisation to particular regions within the same membrane,^38^ can lead to substantially higher local concentrations than the total cellular fraction would imply. Phosphoinositides also exhibit leaflet asymmetry, with e.g. PI(4,5)P_2_ being found within the inner leaflet of the plasma membrane, with reports of it constituting up to 5% of total lipids.^8,46^ While the outer leaflet of human plasma membranes is abundant in enormous lipids with complex glycosylation patterns,^47,48^ within the inner leaflet it is phosphoinositides which bear one of the largest headgroups relative to other phospholipids, which protrude beyond the membrane surface into solution. They are also the most negatively charged of the major plasma membrane inner leaflet lipids. These two properties in particular set them apart and confer a suite of biochemical interactions not available to other phospholipids. During apoptosis this plasma membrane asymmetry is disrupted, and phosphoinositides have been reported to be present in elevated levels within the outer leaflet where they may act as ‘eat-me’ signals in cell death.^49^ Although not canonically referred to as phosphoinositides, membrane lipids containing the core phosphatidylinositol structure have been seen in other domains of life, for example in mycobacteria.^50,51^

### Computational approaches for studying phosphoinositides

A variety of computational approaches are available to learn something about the interactions and dynamics of biomolecules. These include docking,^52,53^ artificial intelligence (AI) methods,^54–57^ quantum mechanics/molecular mechanics (QM/MM) approaches,^58,59^ and molecular dynamics (MD) simulations.^60,61^ Amongst these, MD simulation has shown particular utility in the study of lipid–protein interactions.^62–65^ At its core, MD simulation is a physics-based method to predict how a group of particles (representing the biomolecule(s) of interest) propagate through time and space, using classical mechanics and an underlying potential energy function which describes particle interactions. The functional form of this potential energy function and the set of parameters associated with it are termed the ‘force field’. Parameters for a given biomolecule are obtained from experiments and quantum mechanical calculations. The successful application of the simulation relies on having a well-parameterised model and sufficient sampling of conformational space, coupled to appropriate simulation design and analysis. Common force field choices for membrane protein systems include CHARMM,^66,67^ AMBER,^68–70^ and MARTINI.^24,71^ Simulations may be performed at all-atom resolution, or at ‘coarse-grained’ (CG) resolution, in which groups of atoms are represented as single particles. This is the case for the popular MARTINI force field,^24^ which has shown particular utility in simulation of membrane systems and lipid-protein interactions.^25,64,72,73^ CG resolutions decrease the computational load of the calculations and allow greater sampling of time and space. The compromise on atomic detail may be tempered by conversion^74,75^ of the end-point of a CG simulation back to all-atom detail followed by subsequent all-atom simulation, i.e. multiscale simulation.^76^ Other approaches to addressing the sampling challenge include the use of special purpose supercomputers such as ANTON,^77^ and a group of techniques collectively termed ‘enhanced sampling’,^78^ which are essentially statistical mechanical and algorithmic tricks to enforce sampling of certain regions of space or ‘collective variables’. This review will share examples of all three of these strategies as it pertains to simulations of phosphoinositides.

Amongst phospholipids, phosphoinositides represent one of the more challenging lipid groups for MD simulation due to their highly charged headgroups. For example, while the net headgroup charge of PI(4,5)P_2_ lipids has been estimated as ca. 4 by ^31^P NMR and QM methods in simplified systems,^41,79^ the magnitude and charge distribution may vary substantially *in vivo* depending on physiological microenvironment.^80,81^ This last part is key, as a PI(4,5)P_2_ lipid in the vicinity of a cluster of basic residues may be induced toward the −5 state, while in other cellular scenarios may adopt forms with reduced net charge. The vast majority of simulation approaches employ a fixed charge model in which charge states are set at the beginning of the simulation and remain thus. The choice of charge state is therefore non-trivial. While this is a general challenge in MD simulations,^82^ it is perhaps more acute for certain phosphoinositides. Though -4 variants of PI(4,5)P_2_ are most common,^83^ -5 variants have been seen.^84^ What is most appropriate depends on the system in question and testing of multiple variants may be necessary. Despite these challenges, significant effort has been put into the continued improvement of phosphoinositide parameters, and upgraded versions continue to be released.^83,85,86^ This even extends beyond the core group of mammalian phosphoinositides to more exotic forms with additional modifications, such as those found in *Mycobacterium tuberculosis*.^87^

A theme which will become apparent in the course of this review is that amongst the various phosphoinositide species, PI(4,5)P_2_ has received particularly high levels of attention in simulation and structural studies. This may in part correlate with its inherent biological importance and its relative abundance under a range of cellular states. It may also reflect its localisation to (the inner leaflet of) the plasma membrane, where much membrane protein research has historically tended to focus, especially from a pharmacological perspective. Pragmatic factors such as the early availability of parameters and seminal simulation works on PI(4,5)P_2_ may also have contributed.^38,84,88,89^ We note that simulation parameters are now available for all phosphoinositides species in both all-atom and CG resolutions, and continue to be improved.^83,85,90,91^

More generally, parameters now exist for many thousands of different membrane lipid species.^86,92,93^ This explosion in parameter space has been coupled to knowledge from experimental lipidomics^27,29^ to allow simulations in complex asymmetric membranes which mimic e.g. cell-type specific plasma membranes^94,95^ and other subcellular membranes.^25,96–98^ The mutual lipid-lipid interplay between species within complex biologically relevant membranes can be critical to the biological activity of phosphoinositides^99^ and the potential to capture this in simulations is a powerful ability. In addition to improvements in parameter quality, new tools for the setup and analysis of simulations continue to be developed.^93,100–105^ In particular, specialised tools for the analysis of lipid-protein interaction have emerged, including PyLipID^106^ and ProLint,^107^ as well as refinement of more advanced simulation methodologies such as calculating Δ Δ Gs for lipid binding.^108^

These specific advances have occurred to the continual background march of hardware improvement^109^ and general force field quality.^24^ Notably, the widespread use of GPU computing and the corresponding updates to simulation code have dramatically increased the timescales accessible to research groups.^110^ CG simulations of small membrane proteins such as GPCRs may now reach hundreds of microseconds without breaking too much of a sweat, while length scales of hundreds of nanometres have also been seen.^111–116^ On commodity compute devices atomistic simulations are comfortably of the order of several microseconds for most groups and system sizes, while special purpose devices may routinely reach hundreds of microseconds as a matter of course.^117^ The ‘dawn’ of exascale computing^118^ will further increase accessible time and length scales.^119^

Coupled with new experimental capabilities in structural biology^18^ and biophysics,^120^ these improvements in simulation approaches have been applied alongside experiments by hundreds of scientists to unlock the molecular basis for many exciting new facets of phosphoinositide biology. In the following section we highlight a range of case studies. We then draw it together to summarise an emerging picture of simulation-based analysis of phosphoinositide influence on membrane protein structure, function, and dynamics. We end by taking a critical look at challenges and imminent advances.

## Recent Case Studies

### G-protein-coupled receptors

GPCRs constitute the largest family of human membrane proteins^121^ and play key roles in signal transduction at the plasma membrane,^122^ and at subcellular membranes.^123–125^ The core unit of these receptors consists of a seven transmembrane helical bundle, which may be differentially decorated with extracellular and intracellular domains.^126^ GPCRs are activated by a diverse array of stimuli including binding of small molecule odorants,^127^ peptide hormones,^128^ neurotransmitters,^129^ and by photons of light.^130^ This leads to a conformational change in the helical bundle, coupling to intracellular interaction partners such as G-proteins and arrestins, and signal propagation to downstream cascades ultimately leading to a cellular outcome.^131^

Membrane lipids bind and control GPCR function via diverse modes. The role of cholesterol has been the subject of particularly extensive study by experiment and simulation.^32,132–134^ Phospholipids have been known to bind and modulate GPCR structure and function for some time.^135–138^ However, it is only relatively recently that phosphoinositide modulation of GPCRs has moved into the spotlight.^22^

**Figure 2:**
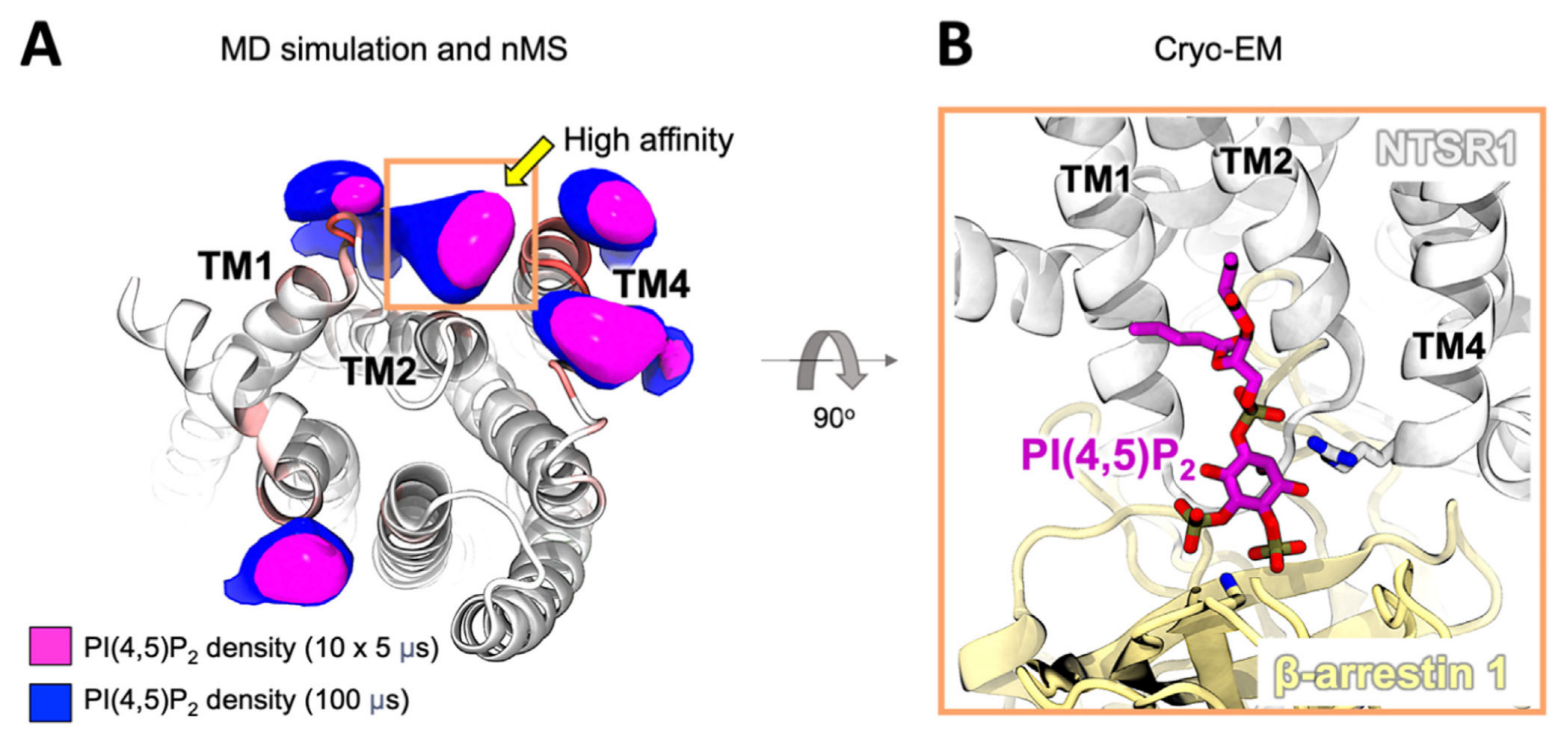
PI(4,5)P_2_ binding sites on the NTSR1 GPCR identified by simulation and experiment. (A) View from the cytosol onto the base on the NTSR1 showing simulation derived densities for PI(4,5)P_2_ around the TM helices.^21^ Densities are shown for an ensemble of short simulations (magenta), and one long simulation (blue) to demonstrate convergence of the simulation over these timescales. The site which showed the highest affinity for PI(4,5)P_2_ in the simulations and nMS is labelled. Helices with red patches indicate residues with high levels of direct phosphoinositide interaction. (B) Side view of a Cryo-EM structure of NTSR1 in complex with β-arrestin 1 showing short-chain dioctyl PI(4,5)P_2_ resolved at the same high affinity site (PDB ID: 6UP7).^140^

An integrated simulation and native mass spectrometry study in 2018 discovered that PI(4,5)P_2_ lipids were able to bind directly to Class A GPCRs and enhance G-protein coupling.^21^ In this work, the interactions of PI(4,5)P_2_ with the Neurotensin receptor 1 (NTSR1), the Adenosine A_2A_ receptor (A_2A_R), and the β_1_ adrenergic receptor (β_1_AR) were first detected by structural native mass spectrometry. CG simulations of up to 100 μs duration were then employed to predict the locations of these interactions on the membrane exposed surface of the GPCR. Key residues seen to bind PI(4,5)P_2_ in the simulations were used to inform a mass spectrometry mutagenesis strategy which identified TM4 and TM1 as the major PI(4,5)P_2_ binding region on NTSR1, alongside several lower affinity sites (Figure 2A). Interestingly, in both simulations and mass spectrometry experiments, PI(4,5)P_2_ outcompeted all other phosphoinositide sub-types, including the more negatively charged PI(3,4,5)P_3_. This is indicative of specific complementary geometries and not only simple correlation with net headgroup charge. High-throughput CG simulations then expanded this analysis to nine other Class A GPCRs, demonstrating conserved PI(4,5)P_2_ interaction, albeit with variability in the precise interaction pattern. The functional consequences of this interaction were demonstrated by employing potential of mean force (PMF) simulations^139^ in which a mini-G-protein was pulled away from the A_2A_R under ±PI(4,5)P_2_ conditions. This showed a

ca. 50% enhancement in the stability of GPCR-G-protein complex in the presence of PI(4,5)P_2_, which acted as a molecular glue bridging between the GPCR and G-protein. This agreed well with complementary mass spectrometry G-protein coupling experiments which showed selective enhancement of complex formation for mini-Gα_s_ over mini-Gα_i_ and mini-Gα_12_, and withfluorescence-based GTPase functional assays in the presence and absence of PI(4,5)P_2_.^21^ Intriguingly, two years after this discovery, a cryo-EM structure was published of NTSR1 bound to β-arrestin 1.^140^ This structure showed a short-chain dioctyl PI(4,5)P_2_ lipid bound at the same high affinity TM4 site previously identified in the simulations and mass spectrometry (Figure 2B). These combined works uncovered the structural and functional basis for a new biological role for PI(4,5)P_2_ in GPCR signalling, with broad consequences for cell biology.^8,141^ Of particular note, while most known roles of phosphoinositides have their origins in the culture plates and test tubes of cell biology and molecular biology approaches, the present story began with simulations and chemistry at the molecular level within computers and mass spectrometers.

Structural coverage of GPCRs has increased exponentially in recent years.^142^ The number of experimentally determined structures in the PDB and GPCRdb is now well over one thousand.^143^ The advent of predictive AI models such as the AlphaFold suite,^54^ Chai-1,^57^ and RoseTTAFold^56^ provide another means of obtaining initial structural coordinates for simulation,^144–146^ alongside traditional homology modelling.^147^ This has presented new opportunities for molecular simulations. A number of studies have leveraged the increased availability of structures to perform simulations of GPCRs in both active and inactive states to assess the conformational dependence of phosphoinositide interactions.^148–154^ Song et al., identified differences in the free energies of interaction for PI(4,5)P_2_ with binding sites on the A_2A_R between inactive, active, and active-miniG states.^148^ Ma et al., subsequently showed the stiffening effects of PI(4,5)P_2_ on both the inactive and active states of A_2A_R in limiting the accessible conformational landscape.^151^ Later experiments employing ^19^F NMR in nanodiscs suggested that in addition to the role of PI(4,5)P_2_ in enhancing G-protein coupling,^21^ it may also increase population of the A_2A_R active state in the absence of G-protein.^155^ CG simulations combined with experimental measurements have reported similar PI(4,5)P_2_ induced stabilisation of the active state of the Ghrelin receptor,^154^ as well as an influence on its coupling to β-arrestin 1.^156^ Most recently PI(4,5)P_2_ interaction with ICL3 has been suggested to stabilise the active state of β_2_AR.^153^

Schiøtt and colleagues performed detailed work on the Class B Glucagon receptor (GCGR), finding that in contrast to Class A GPCRs, PI(4,5)P_2_ preferentially bound the inactive state of the receptor.^150^ Interestingly, the authors also found that binding was modulated by acyl tail saturation pattern, supported by native mass spectrometry measurements. This is a novel observation as phosphoinositides *in vivo* have particularly uniform acyl tail composition of the stearoryl/arachidonoyl form,^42^ and most interactions seen in simulations and structures tend to be dominated by headgroup electrostatics, with tails being dynamic and/or unresolved due to inherentflexibility and the use of short-chain derivatives.^17^ An influence of acyl tail saturation pattern on PI(4,5)P_2_ clustering propensity has also been reported in simulations in the absence of protein.^157^

Amongst Class F GPCRs, CG simulations of the hedgehog signal transducer Smoothened (SMO) predicted PI(4,5)P_2_ encounter complexes.^158^ This is intriguing as SMO signalling is intricately linked to primary cilia, a region which contains special zones of phosphoinositide enrichment.^159^ Joubert’s syndrome, a human ciliopathy characterized by impaired Hh signaling and human birth defects, can be caused from mutations in a 5-positon phosphatase (Inpp5e) which lead to alterations in the distribution of ciliary PI(4,5)P_2_.^12,159–161^

As well as focussed simulations of different conformational states of particular GPCRs, simulations may also assess trends across larger groups. Sejdiu and Tieleman performed simulations of 23 unique GPCRs and characterised their ‘lipid fingerprint’, that is, the unique lipid interaction profile of each GPCR.^149^ While phosphoinositide species interacted with all 23 GPCRs, the location and strength of interaction showed substantial variation. Most recently, CG simulations of 15 human Class B1 GPCRs were performed in both active and inactive states.^162^ A conserved state-dependent pattern of PI(4,5)P_2_ interaction was seen across the subfamily at the TM6/7 – H8 interface, in agreement with earlier CG simulations of GCGR.^150,152^ This observation is particularly intriguing as two antagonists of GCGR have been structurally resolved bound at this same interaction site (PDB ids: 5EE7 and 5XEZ).^163,164^ A positive allosteric modulator (PAM) used to stabilise structures of glucose-dependent insulinotropic polypeptide receptor (GIPR) also binds adjacent to this site, and has been used to stabilise the receptor in complex with the antidiabetic therapeutic tirzepatide (Mounjaro) (PDB id: 7RBT).^165^ This raises the intriguing prospect of possible direct phosphoinositide / drug competition and synergistic binding effects at this region.

**Figure 3:**
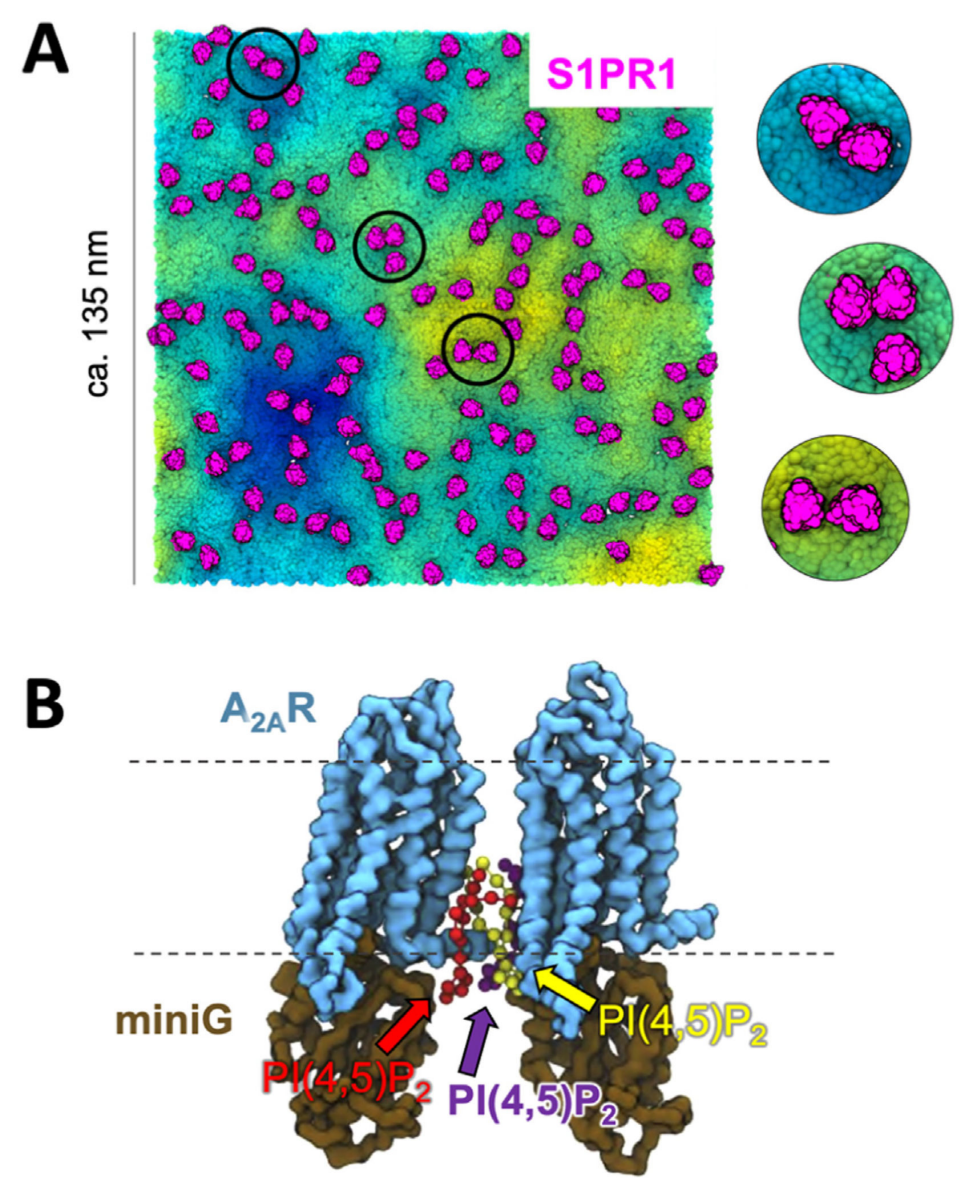
Phosphoinositides can mediate lateral GPCR interactions. (A) 144 S1PR1 GPCRs (pink) in a membrane with complex asymmetric lipid distribution mimicking that of a mammalian plasma membrane. The image shown is from the end of a 10 μs CG simulation. (B) An A_2A_R dimer with interfacial PI(4,5)P_2_ lipids mediating the interaction. We thank Dr. Heidi Koldsø and Dr. Wanling Song for the figure. Figure (adapted) reprinted with permission from 166 and 168. Copyright (2015) American Chemical Society.

Phosphoinositide interactions with GPCRs have also been modelled in large membrane patches measuring up to ca. 135 nm across and containing 144 GPCR proteins embedded in plasma membrane like lipid compositions^166–168^ (Figure 3A). As well as providing excellent statistics on phosphoinositide behaviour, these simulations allow assessment of *in vivo* like factors including e.g. competition effects between different phosphoinositide species such PI(4,5)P_2_ and PI(3,4,5)P_3_, the involvement of membrane curvature, and of lateral protein–protein interactions. In this spirit, clustering of PI(4,5)P_2_ around the S1P receptor 1 (S1PR1)^166^ and NTSR1^167^ have been observed. Similarly, work on the A_2A_R embedded in complex membranes employed simulations coupled to Markov state modelling to predict PI(4,5)P_2_ mediated enhancement of A_2A_R oligomerisation^168^ (Figure 3B).

A common thread in all simulation studies of GPCRs in multicomponent membranes, is that despite comprising a small percentage of the simulated lipid composition, phosphoinositides routinely outcompete all other inner leaflet phospholipids for interaction with the GPCR. This is especially pertinent for lipid species which, like phosphoinositides, have net anionic headgroup charges such as phosphatidylserine (PS) and phosphatidylglycerol (PG) and which have previously been reported to modulate GPCRs.^135,169–171^ This highlights the importance of including the biologically relevant phosphoinositide species in experiments and simulations investigating interactions of lipids with GPCRs. It also raises the question of how many anionic phospholipid interaction sites and functional effects previously reported from *in vitro* and *in silico* studies of GPCRs in simple membranes without phosphoinositides may be abrogated or enhanced by phosphoinositides *in vivo*.

### Ion channels

Ion channels have a particularly close relationship with phosphoinositides.^35^ This relationship has been subject to extensive structural, functional, and computational study.^172–174^ We focus on transient receptor potential (TRP) channels, for which phosphoinositide modulation is diverse and emerging, and on inward-rectifying potassium (Kir) channels, which require PI(4,5)P_2_ for activation.

#### Transient receptor potential channels

**Figure 4:**
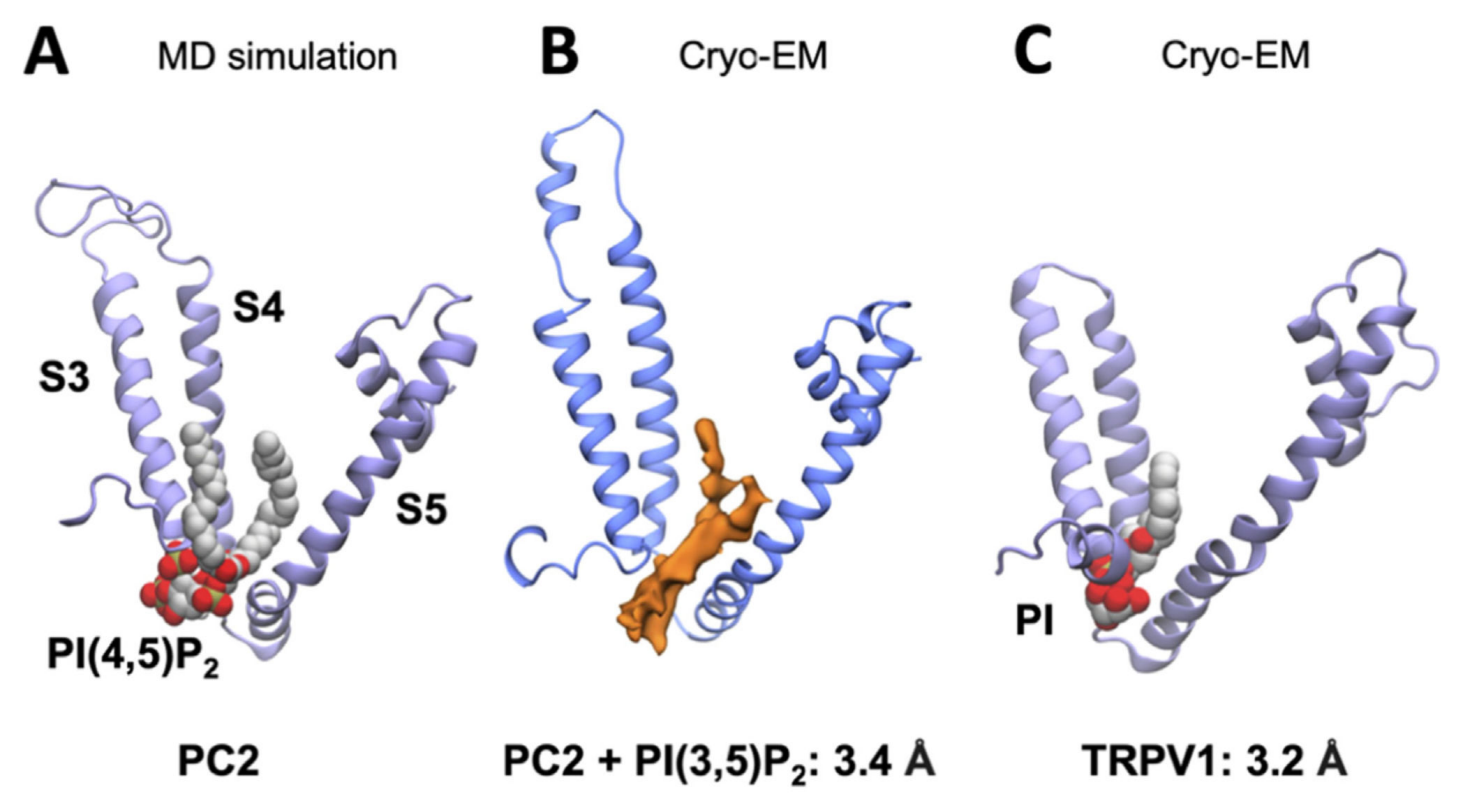
A phosphoinositide binding site on PC2. (A) CG simulation-based identification of a PI(4,5)P_2_ binding site between helices S3/4/5 of PC2.^181^ (B) Lipid density seen at the same site in a cryo-EM structure of PC2 solved in the presence of PI(3,5P)_2_ (PDB ID: 6T9O, EMDB: 10419).^181^ (C) Phosphatidylinositol bound to the same site in a cryo-EM structure of TRPV1 (PDB ID: 5IRZ).^20^ Figure modified from 181.

Transient receptor potential (TRP) channels are a major class of tetrameric ion channel,^175^ and their dysfunction has been implicated in a range of diseases.^176–178^ TRP channels have been shown to be functionally modulated by lipids including sterols and phospholipids.^179^ The binding of phosphoinositides to TRP channels has been explored in a range of structural and simulation studies.^20,180–186^ In 2020, a combined simulation, biochemical, and structural study explored the binding of a number of phosphoinositide species to polycystin-2^181^ (PC2; also known as TRPP2 or PKD2). Cryo-EM structures of PC2 in the presence of PI(3,5)P_2_ (to 3.4 Å), and of PI(4,5)P_2_ (to 3 Å) revealed non-protein density between the transmembrane helices S3, S4 and S5 (Figure 4). As is often the case in structural studies, lipid identity could not be unambiguously assigned from the experimental electron density alone. The authors addressed this challenge by performing CG simulations of PC2 in lipid bilayers, in which they observed spontaneous binding of PI(4,5)P_2_ lipids to the same S3, S4, S5 region seen in the PC2 cryo-EM structures (Figure 4A,B). This region had also previously been seen to bind PI, PI(4,5)P_2_, and vanilloids (e.g. capsaicin) in TRPV1/5 cryo-EM structures and MD simulations of TRPV6.^20,180^ Gao et al., noted in their phosphoinositide bound structure the possibility of the pocket being suited to bind a range of phosphoinositide species.^20^ Simulations designed to calculate free energies of lipid association with this site on PC2 from the PMF^139^ well-depth obtained values of ca. −9 kJ/mol (PS), −20 kJ/mol (PI), −28 kJ/mol (PI(3,4,5)P_3_), and −37 kJ/mol (PI(4,5)P_2_). While this suggested a specific interaction of PIP_2_ over PS, PI, and PI(3,4,5)P_3_, the authors noted the limitations of the CG simulation model in distinguishing between the PI(4,5)P_2_ and PI(3,5)P_2_ isoforms. A biochemical assay in the form of ‘PIP strips’ provides one route to experimental interrogation of lipid selectivity.^187,188^ This assay consists of a nitrocellulose membrane dotted with different lipid species. Binding of the protein of interest to these lipid spots can then be detected using antibodies. Wang et al., employed this approach for PC2 in detergent micelles to demonstrate selective binding of phosphoinositide species over other lipids (PA, PS, PE, S1P), in agreement with the simulations.^181^ However high variation between biological repeats circumscribed further dissection of selectivity between phosphoinositide species.

Delling and colleagues recently applied detailed electrophysiology and simulation work to demonstrate binding of a ciliary-enriched oxysterol (7β,27-DHC) to PC2 at a site which included the S4-5 linker, and functional modulation of channel current.^182^ This oxysterol site overlapped with the putative phosphoinositide binding pocket previously identified from the structures and simulations.^20,181^ Remarkably, inclusion of either PI(3,5)P_2_ or PI(4,5)P_2_ decreased the modulatory effects of the oxysterol on PC1/2 current density, while PI(4)P completely abolished channel activation.^182^ Taken together, the high affinity competition indicated both the veracity of the previously identified phosphoinositide binding pocket, and suggests PI(4)P may be the key biologically relevant phosphoinositide species in controlling PC2 channel function at this site. This finding aligns with the cellular localisation of PC2 channels to cilia membranes, a region enriched in PI(4)P lipids.^159^

These studies highlight both the technical challenges of interrogating phosphoinositide interactions with membrane proteins, and how these challenges may be met by an integrated approach combining simulation and experiment. The paradigm of using CG simulation to assign molecular identity to putative lipid density in cryo-EM structures was recently developed into the LipIDens tool.^23^

In addition to phosphoinositide interaction sites with defined tertiary structure, interactions with intrinsically disordered regions (IDRs) of membrane proteins have been observed^189,190^. In the context of ion channels a fascinating example is provided by the ca. 150 residue N-terminal IDR of TRPV4.^191^ Goretzki et al., integrated a suite of experimental approaches and multiscale simulations to map the molecular level detail of PI(4,5)P_2_ interaction with the IDR ensemble, and its relationship to channel activity. CG simulations combined with NMR experiments delineated a mechanism whereby interaction of PI(4,5)P_2_ with a particular region of the IDR led to a mechanical pull-force on the ankyrin repeat domain (ARD), which ultimately led to force transduction to the TRPV4 core. The extent and structural basis for phosphoinositide selectivity when it comes to membrane-proximal IDR ensembles is intriguing to consider, and simulations combined with machine learning technologies are increasingly well positioned to further address this.^192,193,66^

#### Inward rectifying potassium channels

Potassium channels facilitate the selectiveflow of K^+^ ions across cellular membranes. These tetrameric ion channels are one of the best characterised and most ubiquitously expressed integral membrane proteins with wide ranging roles in physiology and disease.^194^ The three main groups of potassium channels are inward rectifying (Kir), voltage-gated (Kv), and two-pore (K2P) potassium channels. A variety of structural^17,195^ and functional^196,197^ studies have established central roles for lipids in potassium channel biology. A particularly intimate relationship is seen between Kir channels and PI(4,5)P_2_ lipids, which act as channel activators.^198^ This section highlights some of the key ways in which simulations have enriched our understanding of various aspects of phosphoinositide biology as it relates to Kir channels, as well as remaining challenges.

**Figure 5:**
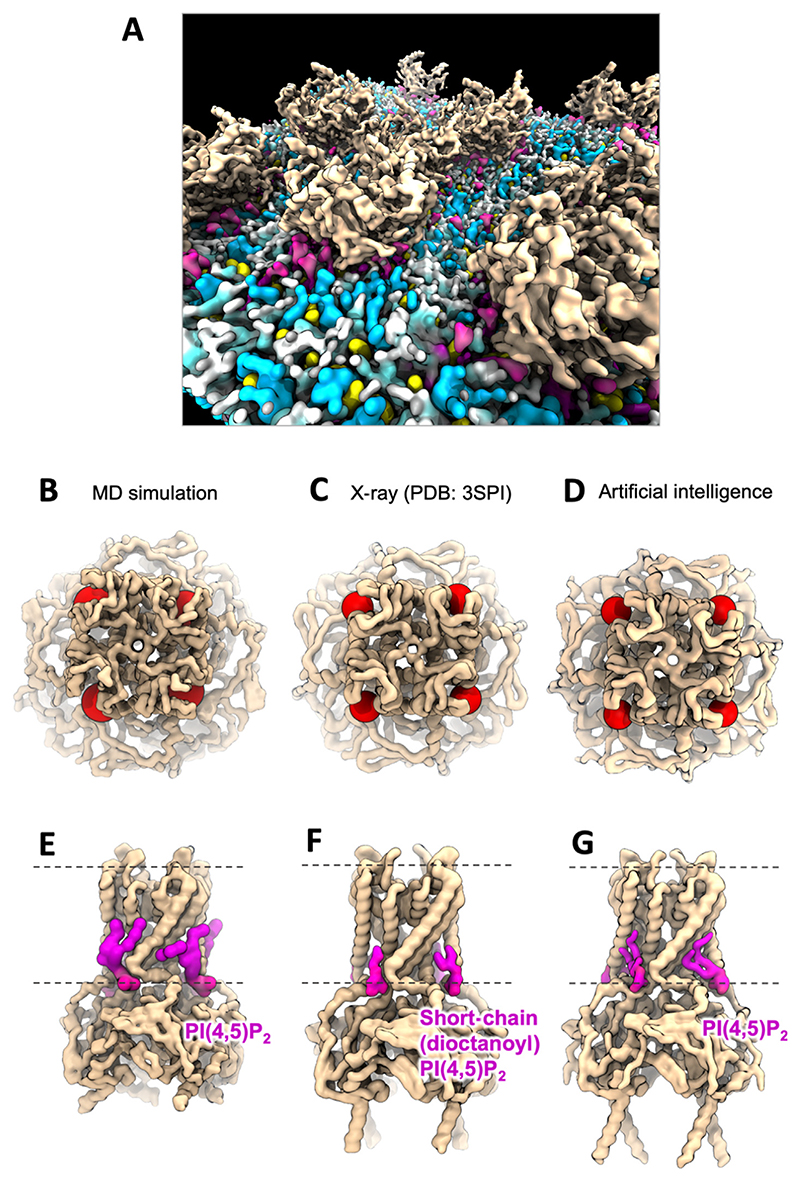
Primary PI(4,5)P_2_ binding site on Kir2.2 from simulation, experiment, and AI. (A) View of the inner leaflet surface from a simulation containing multiple Kir2.2 channels embedded in a complex and crowded membrane environment.^199^ PI(4,5)P_2_ (magenta) is clustered around the channels (beige). (B) The primary PI(4,5)P_2_ sites identified on homotetrameric Kir2.2 by CG MD simulation.^84,199^ (C) the same site identified by X-ray crystallography (PDB id: 3SPI),^17^ and (D) by AI. The AI coordinates shown were predicted using Chai-1^57^ (https://lab.chaidiscovery.com/), the monomeric FASTA sequence from PDB id: 3SPI, and the PubChem^202^ SMILES string for PI(4,5)P_2_. The red spheres denote the position of the phosphate group bridging the glycerol and inositol moieties of PI(4,5)P_2_. The perspective shown is from the extracellular side of the bilayer. (E–G) Lateral view of the same structures, with the PI(4,5)P_2_ lipids shown in magenta. Dotted lines denote the approximate position of the bilayer – solvent interface. We thank Dr. Anna Duncan for providing simulation coordinates used to produce the figure.

At the macroscale, it is now possible to simulate large patches of membrane containing hundreds of proteins embedded in complex membrane compositions.^94,111^ Thus Duncan et al., performed CG simulations of membrane patches containing >100 Kir2.2 channels in a plasma membrane like lipid composition^112^ (Figure 5A). Simulations under a range of defined lipid regimes and protein concentrations revealed a role for PI(4,5)P_2_ in influencing channel crowding and bilayerfluctuations. Follow up work assessed the nanoscale interactions of PI(4,5)P_2_ with Kir2.2^199^ by leveraging the large complex membranes to gather sufficient statistics to assess competition effects between different lipids. PI(4,5)P_2_ was seen to bind at the experimentally determined primary site on Kir2.2^17^ (Figure 5). Interactions at a secondary^200,201^ anionic lipid site were also observed. Though PI(4,5)P_2_ interaction was most favourable over other lipid species at both sites, anionic PS lipids (net headgroup charge -1) were able to bind if the PI(4,5)P_2_ concentration was low enough. The molecular basis of PI(4,5)P_2_ selectivity over other phospholipids resulted from protrusion of the comparatively larger inositol headgroups into solution, with the 4′ and 5′ phosphoryl moieties able to interact with key basic residues of the cytoplasmic domains which were beyond the reach of phospholipids with smaller headgroups. Thus in this case selectivity resulted both from structure and from charge. Free energy perturbation (FEP) simulations were applied to estimate the ΔΔ G for transforming PI(4,5)P_2_ to PS at both lipid binding sites. The calculations showed the selectivity for PI(4,5)P_2_ over PS was significantly greater at the primary interaction site (ΔΔG = 43 k J/mol) than at the secondary interaction site (ΔΔG = 29 kJ/mol). Notably, the magnitude of the unfavourable ΔΔG for the secondary site was reduced from 29 kJ/mol to 17 kJ/mol if a PI(4,5)P_2_ lipid was also present within the primary site. That is, PS interaction at the secondary site became more favourable if a PI(4,5)P_2_ lipid was also present within the primary site. This delicate interplay between the two sites may provide the cell with an additional means of differentially modulating channel function by controlling the relative local abundance of competing lipid species.^199^

The location of the primary PI(4,5)P_2_ interaction site characterised by Duncan et al., was first predicted in simulations of KirBac1.1, a Kir3.1-KirBac1.3 chimera, and a homology model of Kir6.2 performed in 2009.^84^ This early work employed CG simulations of PI(4,5)P_2_ in simple membranes containing phosphatidylcholine (PC) and PI(4,5)P_2_. Simulations of 5 μs duration were sufficient to observe spontaneous and sustained binding of PI(4,5)P_2_. Key clusters of basic residues which formed the binding site for the inositol head-group had previously been suggested to be involved in Kir2.1 interactions with PI(4,5)P_2_.^203–205^ A subsequently solved Kir2.2 crystal structure showed short-chain (dioctanoyl) PI(4,5)P_2_ density at the same site (Figure 5). Interestingly, in this case lipid binding seemed robust both to the acyl tail composition/absence, and the use of non-eukaryotic structures and homology models. Further simulation studies of Kir2.2 have continued to support the veracity of the PI(4,5)P_2_ site.^206–208^

Interestingly, this site shows excellent agreement with an AI model we predicted for this review using the Chai-1 web interface^57^ (https://lab.chaidiscovery.com/), and only the primary sequence of the Kir2.2 monomer and the SMILES string for PI(4,5)P_2_ as input (Figure 5D, G). The authors of this model report similar capabilities to AlphaFold3, and the web tool places no limitations on small molecule types which may be assessed. The emerging ability of AI models to predict protein structures in complex with small molecules, including lipids, is likely to be a major accelerator for phosphoinositide research, providing initial coordinate sets from which to launch dynamical physics-based simulation and experimental investigations.

**Figure 6:**
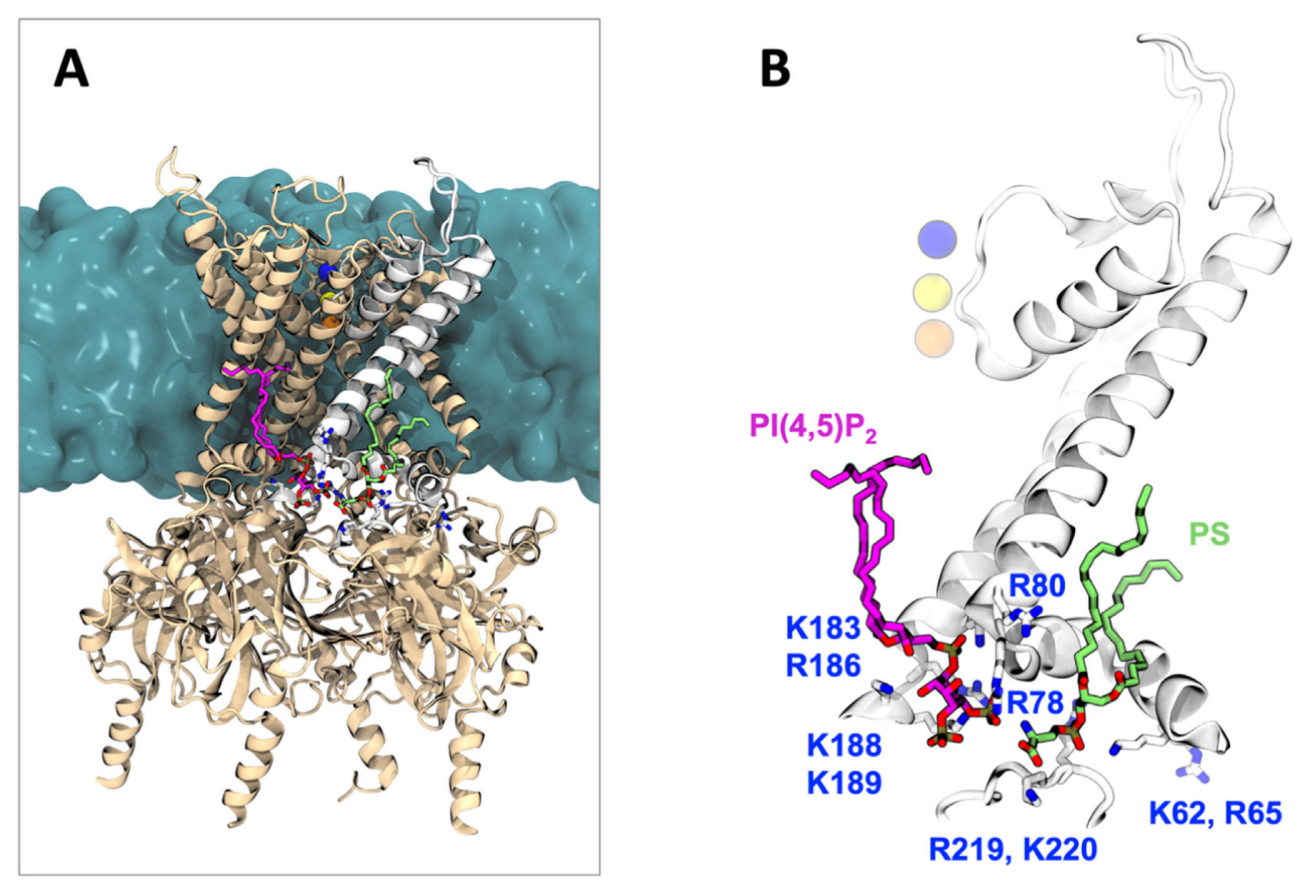
Atomic architecture of a PI(4,5)P_2_ binding site on Kir2.2.^214^ (A) Cross-section of an ANTON simulation snapshot showing a PI(4,5)P_2_ lipid (magenta) binding site on tetrameric Kir2.2 (beige) embedded in a lipid bilayer (blue surface). An adjacent PS lipid is shown in green. (B) Zoomed in view on the PI(4,5)P_2_ site showing how the headgroup is cradled by basic residues during the simulation. K^+^ ions within the selectivity filter are shown in the background as blue, yellow, and orange spheres. We thank Dr. Vishwanath Jogini, Dr. Morten Ø. Jensen, and D. E. Shaw Research for providing coordinates used to produce the figure.

The functional consequences of PI(4,5)P_2_ binding for gating and Kir channel conductance have been explored in a range of simulations works.^209–213^ Tour de force simulations from the D. E. Shaw Research group recently further defined the mechanism of PI(4,5)P_2_ activation of Kir2.2 in atomic detail.^214^ Starting from a closed-state structure,^17^ Jogini et al., introduced a previously characterised mutation at the bundle crossing to obtain an open conducting state, capture key conformational changes at the main activation gate and cytoplasmic domain, and demonstrate the dynamical basis for PI(4,5)P_2_ stabilisation of the open-state in all-atom detail (Figure 6). A particularly notable feature of this work is the degree of sampling achieved, with timescales of up to 200 μs reached for an individual simulation, and on aggregate over a millisecond across all simulations. Despite this immense sampling, the authors note that the presence of PI(4,5)P_2_ and bulk PS failed to evoke pore-opening of the WT channel, as would be expected in the event of a sufficiently accurate force field coupled to sufficient sampling. This suggests that while substantial progress continues to be made, there remains scope for development of all-atom MD in capturing certain mechanistic aspects of phosphoinositide biology occurring on long timescales.

Further studies have leveraged MD simulations to assess the molecular detail of phosphoinositide interaction in the context of Kv channels,^215–221^ and K2P channels.^222,223^ Other examples of phosphoinositides controlling non-K^+^ ion channel structure and function include the calcium-dependent chloride ion channel TMEM16A/ANO1,^224–226^ mechanosensitive channels such as Piezo1,^227^ and voltage-gated sodium channels.^228^ A particularly notable example is the inositol trisphosphate receptor (IP_3_R), where PI(4,5)P_2_ primes the receptor by partially occupying the orthosteric IP_3_-binding site.^229^

### Peripheral membrane proteins

Peripheral membrane proteins (PMPs) exist largely in aqueous solution and interact with the hydrophilic surface of membranes. This is a particularly important group of proteins in relation for phosphoinositide biology, as it encompasses the kinases^230^ and phosphatases^231^ which synthesise and degrade the various phosphoinositide species and thereby regulate their relative abundance and localisation in membranes.^232^ The interactions of PMPs with phosphoinositides is also intimately involved in intracellular signalling cascades, for instance in the enzymatic cleavage of PI(4,5)P_2_ into the second messengers diacylglycerol and inositol-trisphosphate (IP_3_).^233^

Interactions of PMPs with membranes may take a variety of forms. These include binding of a specific lipid headgroup to a specific site on the PMP, more dynamic interactions between patches of complementary charge on the protein surface and the net anionic charge of the inner leaflet, and by insertions of hydrophobic loops or lipidic post translational modifications, e.g. myristoylation.^234^ Structure determination of PMPs in solution or indeed bound to individual lipids is now relatively routine. However, the main biological function of PMPs takes place on the surface of membranes. Stabilising PMPs on membranes to the level required for atomic level structure determination remains challenging.^235^ Simulations have played a critical role in reuniting experimentally determined structures of PMPs with phosphoinositide-containing lipid membranes *in silico* to determine their lipid interactions, binding orientation and dynamics. This is particularly important as the biologically relevant membrane context can influence the conformation of the PMP, and reveal new lipid binding sites.^236^

Extensive all-atom simulations have revealed the molecular basis for how PI(3,4,5)P_3_ drives binding of Bruton’s tyrosine kinase (Btk) to membranes and allosterically stabilises the experimentally determined dimeric form of the protein.^237^ Starting from two copies of the monomeric form of Btk in solution, this study was able to simulate spontaneous binding to PI(3,4,5)P_3_ membranes and Btk dimerization. This impressive feat was achieved through use of the ANTON supercomputer^77^ coupled to aflavour of enhanced sampling simulation termed ‘tempered binding’.^238^

Simulations may also be employed alongside complementary biophysical data to pre-orientate a given PMP on the membrane. Thus in early works in this area Lai et al., used electron paramagnetic resonance (EPR) spectroscopy data to obtain approximate geometries for the C2 domain of Protein kinase Cα relative to PI(4,5)P_2_ containing membranes.^239^ These geometries were used as starting points for simulations, thereby decreasing the level of sampling required compared to *de novo* binding from solution. This approach enabled refinement of C2 domain orientation, determination of lipid:protein stoichiometry, and a molecular level view of key PI(4,5)P_2_ interactions. This same approach was subsequently applied to reveal the molecular basis of stereospecific binding of the GRP1 PH domain to PI(3,4,5)P_3_.^240^

**Figure 7:**
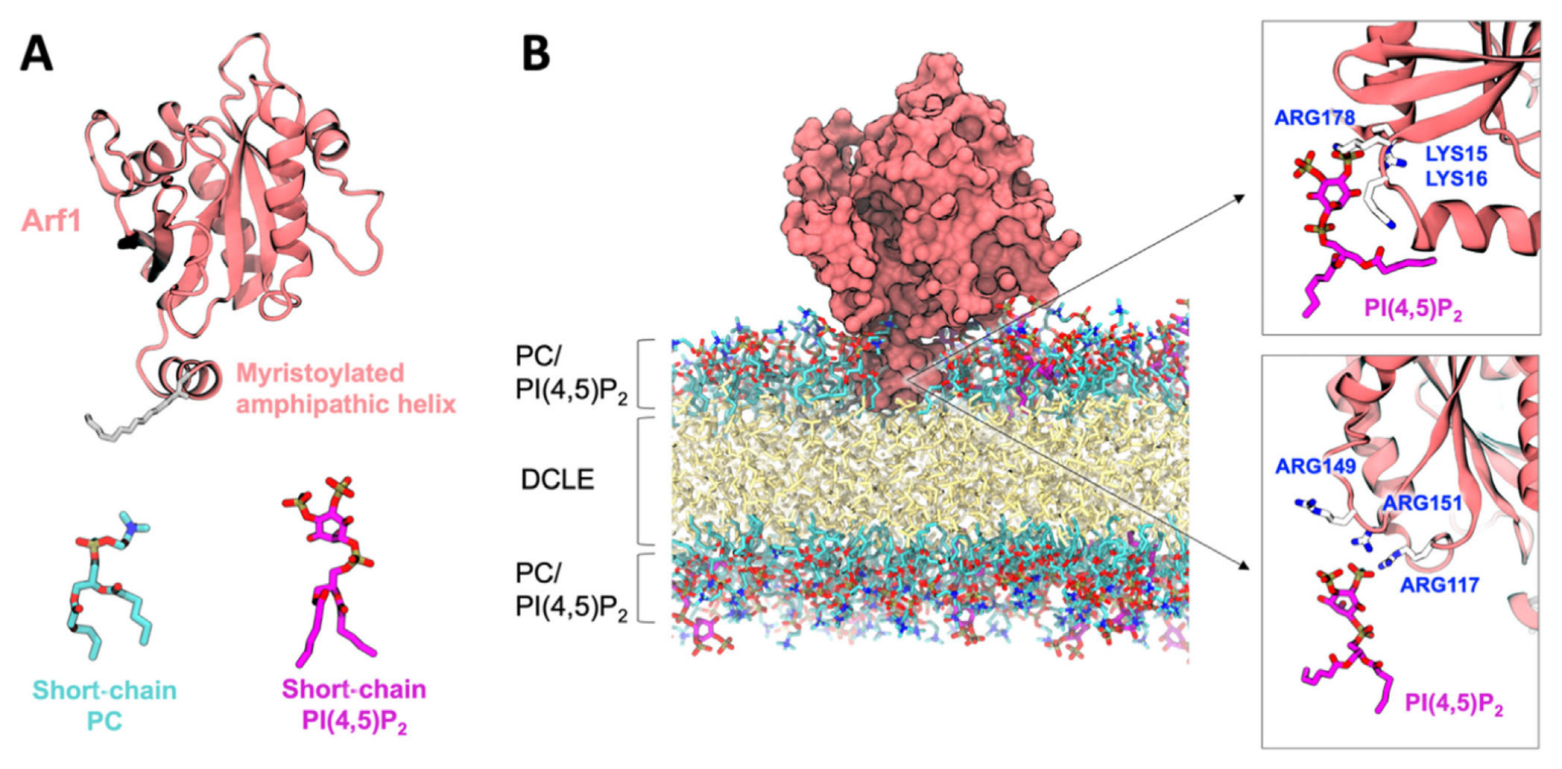
Arf1 membrane interactions.^241^ (A) Structure of myristoylated Arf1 and short-chain PC/PI(4,5)P_2_. (B) Simulation snapshot showing a cross-section of Arf1 bound to a PC/PI(4,5)P_2_ highly mobile membrane mimetic (HMMM) containing a DCLE organic solvent core. Interactions of Arf1 with PI(4,5)P_2_. We thank Dr. Shashank Pant and Prof. Emad Tajkhorshid for providing coordinates used to render the figure.

All-atom simulations of PI(4,5)P_2_ and PI(3,4,5)P_3_ binding to PMPs have also been performed^241–244^ using the highly mobile membrane mimetic (HMMM) model.^92^ This approach replaces the hydrophobic core of the lipid bilayer with organic solvent, atop which phosphoinositide headgroups with shortened acyl tails diffuse. The net effect is to increase the rates of lipid diffusion and therefore enhance the rate at which they explore possible interaction modes with the PMP.^245^ In a recent example of the HMMM approach, Pant and colleagues addressed the orientational dynamics of Arf1 GTPase on PI(4,5)P_2_ membranes^241^ (Figure 7). This particular PMP is localised to the membrane *via* a myristoylated amphipathic N-terminal helix. The increased diffusion rates provided by the HMMM model were leveraged to identify three principle orientational states of myristoylated Arf1 on membranes with different lipid compositions, which were subsequently refined with microsecond all-atom simulations with full lipid representation. Interactions with PI(4,5)P_2_ were key for driving full membrane association and population of these three states, in qualitative agreement with neutron reflectometry (NR) and NMR experiments.^241^

High-throughput CG simulations have been used to assess patterns of phosphoinositide interaction across whole families of pH domain-containing PMPs.^236,246^ Yamamoto and colleagues examined the spontaneous binding of the apo forms of 13 different PH domains to PI(3,4,5)P_3_ and PI(4,5)P_2_ membranes.^236^ Overlay of the simulation derived phosphoinositide interactions showed good agreement with available experimental X-ray crystallographic and NMR structures of the phosphoinositide bound forms of these proteins. In addition to the experimentally known interactions, the simulations also revealed a number of new non-canonical phosphoinositide binding sites for select PH domains. Mutation of key residues within these sites allowed delineation of the main contributors to phosphoinositide binding. For all PH domains, a degree of less specific phosphoinositide clustering around the protein was also indicated to contribute to membrane association. This was consistent with observations from large scale biochemical assessment of 91 PH domains,^247^ and was later supported by free energy calculations for the GRP1 PH domain interacting with PI(3,4,5)P_3_ clusters of varying size,^248^ and by a herculean body of work to extend analysis to 100 different mammalian PH domains.^249^

Further simulation works delineating phosphoinositide interaction with PMPs include on Vinculin,^250^ the Dok7 PH domain,^251^ the K-Ras G domain,^252^ KRas4b,^244^ KRas4b and RAF1,^253^ PLD2,^254^ focal adhesion kinase,^255,256^ PTEN and SHIP2 C2 domains,^257^ PTEN and voltage sensitive phosphatase,^258^ Arf/GEF,^259^ PLCγ1,^260^ Gelsolin,^261^ ORP/Osh proteins,^262–264^ GRP1 PH domain,^265^ murine kindlin-3 PH domain,^266^ BIN1/M-Amphiphysin2,^267^ lymphocyte specific kinase (LCK),^268^ Smurf1 C2 domain,^269^ StarD4,^270,271^ SNX11,^272^ WD repeat domain phosphoinositide-interacting protein 2 (WIPI2),^273^ tubby domains,^274^ and phosphatidylinositol-transfer proteins.^275^ The number of different research groups turning to simulations to interrogate the molecular basis of how their favourite PMP interacts with phosphoinositides is indicative of both the need in this area, and the power of molecular simulation in meeting that need. Notably, while most works to date have dealt with mammalian PMP interaction with PI(4,5)P_2_ and PI(3,4,5)P_3_ lipids, simulations have also shown utility in exploring other phosphoinositide species in non-mammalian contexts, such as PI(4)P interactions with *Plasmodium vivax* Perforin-Like Proteins (PLPs).^276^ Future CG simulations of PMPs are likely to benefit from the GoMartini model of protein structure, which has recently been reported to further enhance the accuracy of conformational modelling of PLCδ1 interacting with PI(4,5)P_2_ bilayers.^277^ For a recent excellent review on the generalized application of molecular dynamics simulations to PMPs broadly.^278^

### Receptor tyrosine kinases

Many fundamental cellular signals are transduced across the plasma membrane by human receptor tyrosine kinases (RTKs).^279^ This family of 58 cell surface receptors share a common molecular architecture consisting of an extracellular ligand binding ectodomain, a single-pass transmembrane (TM) helix, aflexible juxtamembrane (JM) region, and an intracellular protein kinase domain. A variety of additional soluble domains may decorate different types of RTK leading to substantial variety.^280^ Binding of ligands to the ectodomain canonically leads to receptor dimerization, activation of protein kinase activity, and sets in motion a variety of intracellular signalling cascades.^281^ Non-canonical forms of signalling by heterodimerisation of RTKs are also possible.^282^ Phosphoinositides have been shown experimentally to directly bind a range of RTKs,^283^ modulate their function,^34^ as well as control their localisation into high order multimers at the cell surface.^284–286^

**Figure 8:**
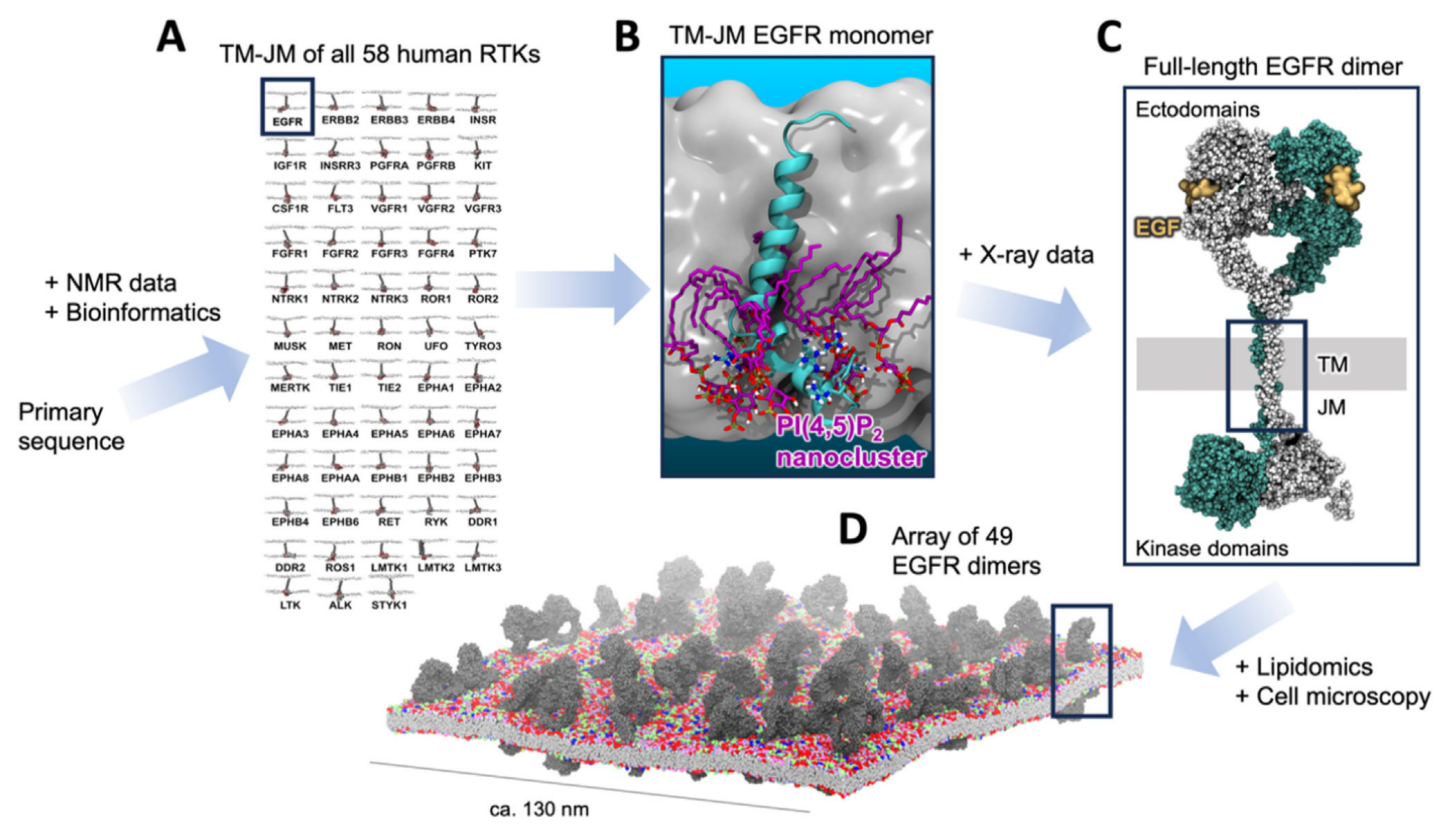
Multiscale simulations of the human RTK superfamily. (A) TM-JM models of all 58 known human RTKs, built from NMR data and secondary structure prediction programs and simulated in 189. (B) A nanocluster of PI(4,5)P_2_ lipids around the TM-JM monomer of EGFR/Erbb1. Image is the endpoint of a 1 μs CG simulation followed by 50 ns of all-atom simulation. (C) Full-length model of the EGFR/Erbb1 dimer built from NMR, X-ray and modelling data. (D) 49 full-length EGFR/Erbb1 dimers embedded in a patch of membrane with a lipid composition approximating that of a mammalian plasma membrane, including inner leaflet PI(4,5)P_2_ and PI(3,4,5)P_3_. The image shown is after 8 μs of simulation. Hedger et al., unpublished.

As major therapeutic targets^287,288^ (e.g. Herceptin against the Erbb2 RTK in the treatment of breast cancer) RTKs have been paid particular attention by structural biologists and atomic resolution structure availability is relatively good for select RTKs^289–291^. However intrinsically disordered regions such as the JM region remain more mysterious, despite their known functional importance.^292^ This is an area where computational approaches are increasingly making impact.^193^ Simulations of the TM-JM constructs of all 58 human RTKs have predicted nanoclustering of PI(4,5)P_2_ around the disordered JM^189^ (Figure 8A, B). This behaviour was driven *via* direct interaction with a conserved polybasic sequence motif found in the region of the disordered JM immediately adjacent to the TM helix. More detailed work focussing on single RTKs including the epidermal growth factor receptor (EGFR),^293^ the tropomyosin receptor kinase A (TrkA),^294^ and the ephrin A2 receptor (EphA2R)^295^ identified similar JM driven PI(4,5)P_2_ interactions. Most recently a combined simulation and NMR study observed this phenomenon for the prolactin receptor JM.^190^ Such nanoclustering of PI(4,5)P_2_ lipids around individual receptors is especially pertinent when it is considered that PI(4,5)P_2_ is a key second messenger substrate in many of the downstream signal cascades which are activated by RTKs. Fluorescence microscopy approaches have indicated that a variety of RTKs are localised into higher order arrays at the cell surface.^284,285,296,297^ If each member of this array has its own local pool of PI(4,5)P_2_ lipids around it, as predicted in the simulations, this would overall add up to a high local concentration of phosphoinositide lipids, with potential implications for signal transduction efficiency and PMP recruitment. CG simulations of large membranes containing tens of full-length RTKs are now feasible, and are expected to shed further light on the molecular architecture of these clusters (Figure 8C, D).

Simulations have also been used to compute the change in free energy for lipid binding to the EGFR TM-JM dimer within PC bilayers.^298^ As expected, PI(4,5)P_2_ binding is significantly more favourable than for other plasma membrane phospholipid species. Notably, the ΔΔG values are of a magnitude similar to those obtained for TM helix dimerization for a range of RTKs.^299,300^ This would seem to lend credence to the idea that one mechanism of PI(4,5)P_2_ modulation of RTK activity may be *via* direct competition with helices and/or modulation of TM dimer crossing angle.^301,302^ Barrera and colleagues subsequently tested this concept experimentally for the EphA2R and identified a marked effect of PI(4,5)P_2_ in promoting dimerization of a particular crossing angle mode.^303^ This is an exciting result which positions helix dimerization as yet another potential microswitch^304^ in phosphoinositide biology. Factors which will be important to address experimentally going forward include the influence of soluble domains, competition between different phosphoinositide species, and the universality or otherwise amongst different RTKs and indeed other single-pass membrane proteins.

**Figure 9:**
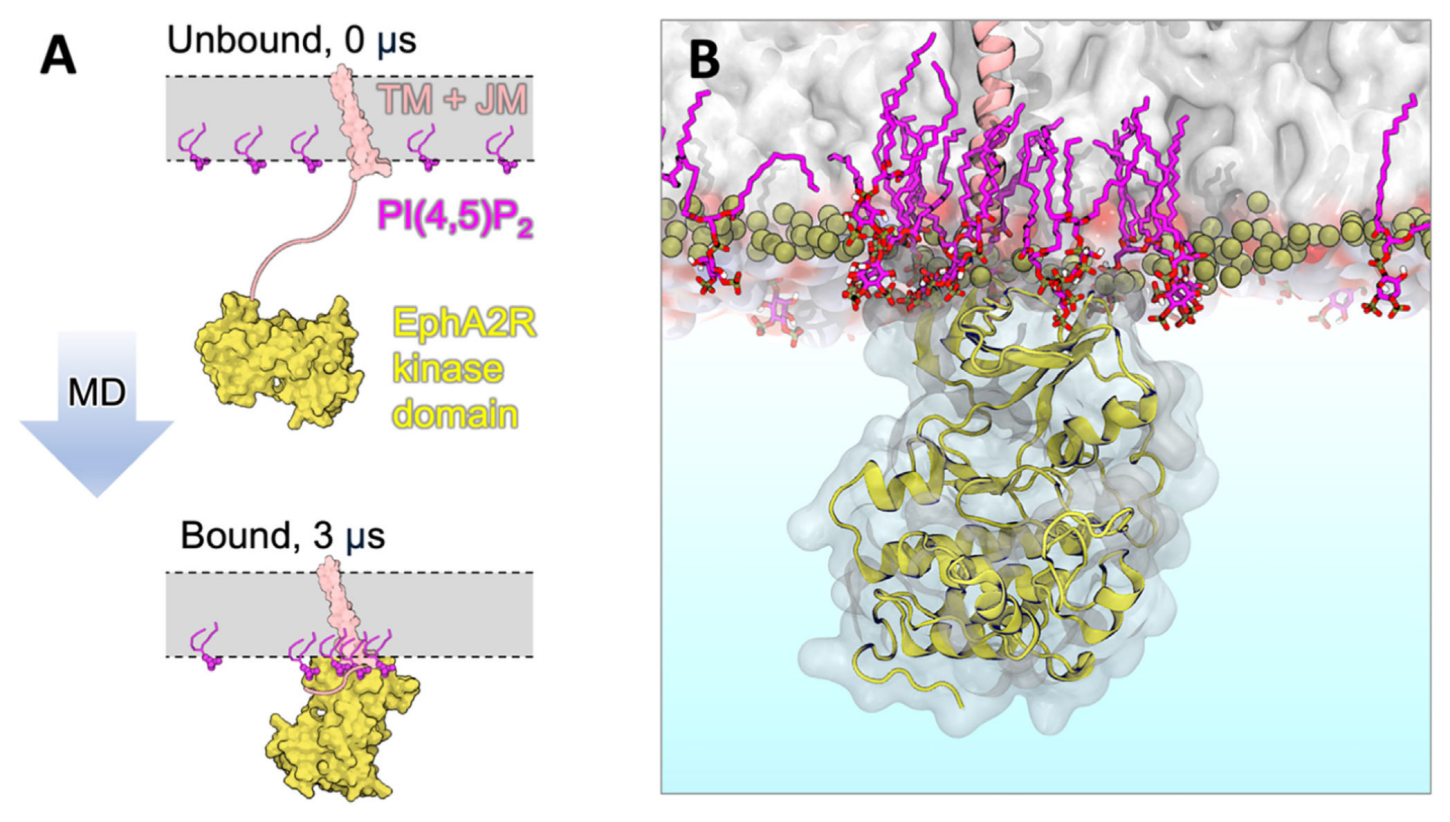
Simulations identify orientations of the EphA2R kinase domain on PI(4,5)P_2_ containing membranes. (A) Schematic of start and endpoint of simulations. (B) Representative snapshot of interface 1 identified in 295. The kinase domain (yellow) is shown interacting with a cluster of PI(4,5)P_2_ lipids (magenta). The JM is located within the cluster. We thank Dr. Matthieu Chavent for the coordinates used to produce the figure.

Other ways in which phosphoinositides influence RTKs include in controlling how the intracellular kinase domains interact with the inner leaflet surface, and recruitment of RTK growth factor ligands to membranes. Chavent et al., used CG simulations to capture the spontaneous binding of the kinase domain to the membrane surface.^295^ The conceptual framework bears resemblance to the PMP – phosphoinositide simulations described in Section ‘**Peripheral membrane proteins’**, albeit in this case with the kinase domain tethered to the membrane by theflexible JM and TM helix (Figure 9). By coupling the simulations to liposome pull-down assays and prior modelling of EphA2R, a key role for PI(4,5)P_2_ was identified in orientating the kinase domains for *trans*-autophosphorylation within EphA2R clusters. Most recently, all-atom simulations have been used to examine heterodimeric EphA2R-EGFR kinase domain interactions with PI(4,5)P_2_.^285^ This study identified intriguing differences between EphA2R and EGFR, in the context of wider experimental work which demonstrated PI(4,5)P_2_ promoted both hetero and homomultimerization of EphA2R and EGFR.

Phosphoinositide species also have key roles in the biology of certain RTK ligands, such as the fibroblast growth factor 2 (FGF2). FGF2 comes into contact with PI(4,5)P_2_ lipids in the inner leaflet during its secretion by the type I ‘unconventional protein secretion (UPS)’ pathway.^305^ PI(4,5)P_2_ interaction induces a fascinating piece of biology in which FGF2 undergoes oligomerisation to form a membrane pore, before exiting the other side of the membrane to arrive in the extracellular space.^306^ Simulations have been applied to capture PI(4,5)P_2_ interactions with FGF2 during this process.^99,307^ Most recently Lolicato *et al*. combined microsecond atomistic simulations with *in vitro* and *in cellulo* assays to address lipid synergy during the initial membrane recruitment of FGF2.^99^ The simulations identified the atomic basis of an intricate lipid interplay in which cholesterol interacted with PI(4,5)P_2_ lipids to increase the inositol headgroup visibility to FGF2 in a manner which resulted in faster binding kinetics and more stable FGF2 – PI(4,5)P_2_ complexes. This is an important finding for phosphoinositide biology generally, as it highlights the consequences of lipid-lipid interplay and complexity for certain phosphoinositide molecular interactions.

### Other membrane proteins

The phosphoinositide interactions of a variety of other membrane proteins have been addressed by simulations. Examples of transporters include PI(4,5)P_2_ modulation of the structure and function of the dopamine transporter,^37,308^ solute carrier spinster homolog 2 (SPNS2),^309^ and SLC4 family transporters.^310–312^ PI(3,5)P_2_ mediated oligomerisation of the endosomal sodium-proton exchanger NHE9,^313^ and phosphatidylinositol stabilisation of purine symporter UapA dimers^314^ have also been addressed with simulations. The influence of phosphoinositides on membrane proteins involved in viral biology,^315–321^ cytoskeleton biology,^322–324^ vesicle trafficking,^325^ autophagy,^273^ and pore formation during programmed cell death,^326^ have also been studied.

### Phosphoinositide clustering

Beyond specific membrane proteins, simulation studies have also addressed the ability of certain phosphoinositides to self-interact and cluster into areas of enrichment in the absence of membrane proteins.^85,327–330^ A key role for divalent calcium has been identified in bridging interactions between the phosphate groups of adjacent PI(4,5)P_2_ lipids.^331^ This effect did not extend to other common inorganic cations nor to PI(3,5)P_2_ lipids,^332^ in qualitative agreement with trends seen influorescence-based spectroscopy and microscopy experiments.^333^ This is particularly interesting in a cell biology context given the role of calcium signalling downstream of many of the signalling cascades in which PI(4,5)P_2_ lipids act as key PMP recruitment factors and second messenger substrates.^283^ Indeed, direct competition between calcium and PMPs for interaction with PI(4,5)P_2_ has been reported^334^. Dynamic modulation of local calcium levels could represent another means of controlling how phosphoinositide species interact with membrane proteins. Acyl tail saturation has also been seen to play a key role in modulating nanocluster formation in CG simulations.^157^

Nanocluster sizes may be increased substantially by the presence of proteins. In a landmark study, van den Bogaart and colleagues addressed the architecture of syntaxin-1A – PI(4,5)P_2_ clusters using super-resolution microscopy and CG simulations.^38^ Microdomains of up to 73 nm in size were experimentally identified in which PI(4,5)P_2_ was the dominant lipid species. Use of phosphatases to knockdown cellular PI(4,5)P_2_ levels is a common trick in phosphoinositide biology, and was applied in this case to demonstrate co-clustering of syntaxin-1A was dependent on PI(4,5)P_2_. Simulations were run in which 64 copies of the transmembrane helix of syntaxin-1A were embedded into a PI(4,5)P_2_:PC:PS membrane. The helices and PI(4,5)P_2_ lipids were seen to spontaneously separate into microdomains knitted together by ionic interactions between negative PI(4,5)P_2_ phosphate groups and basic residues located at the tips of the syntaxin-1A helices, similar to later observations made for RTKs^189^ and the prolactin receptor.^190^ These microdomains remained stable on simulation timescales of up to 6 μs, and could be recreated *in vitro* within giant unilamellar vesicle membranes. Notably, in this case the effects were robust to changes in lipid composition including of cholesterol, which has been seen to influence PI(4,5)P_2_ interactions in other contexts.^99^ Simulations have also played a key role in delineating how phosphoinositide – membrane protein interactions can lead to local remodelling of membranes. BAR domains in particular have been the subject of attention in this area.^267,335–338^ Phosphoinositide interactions with a variety of other remodelling proteins including epsin N-terminal homology domain (ENTH),^339^ cavin1,^340^ and AP180 N-terminal homology domain (ANTH)^341^ have also been studied.

## Summary and Concluding Remarks

**Figure 10:**
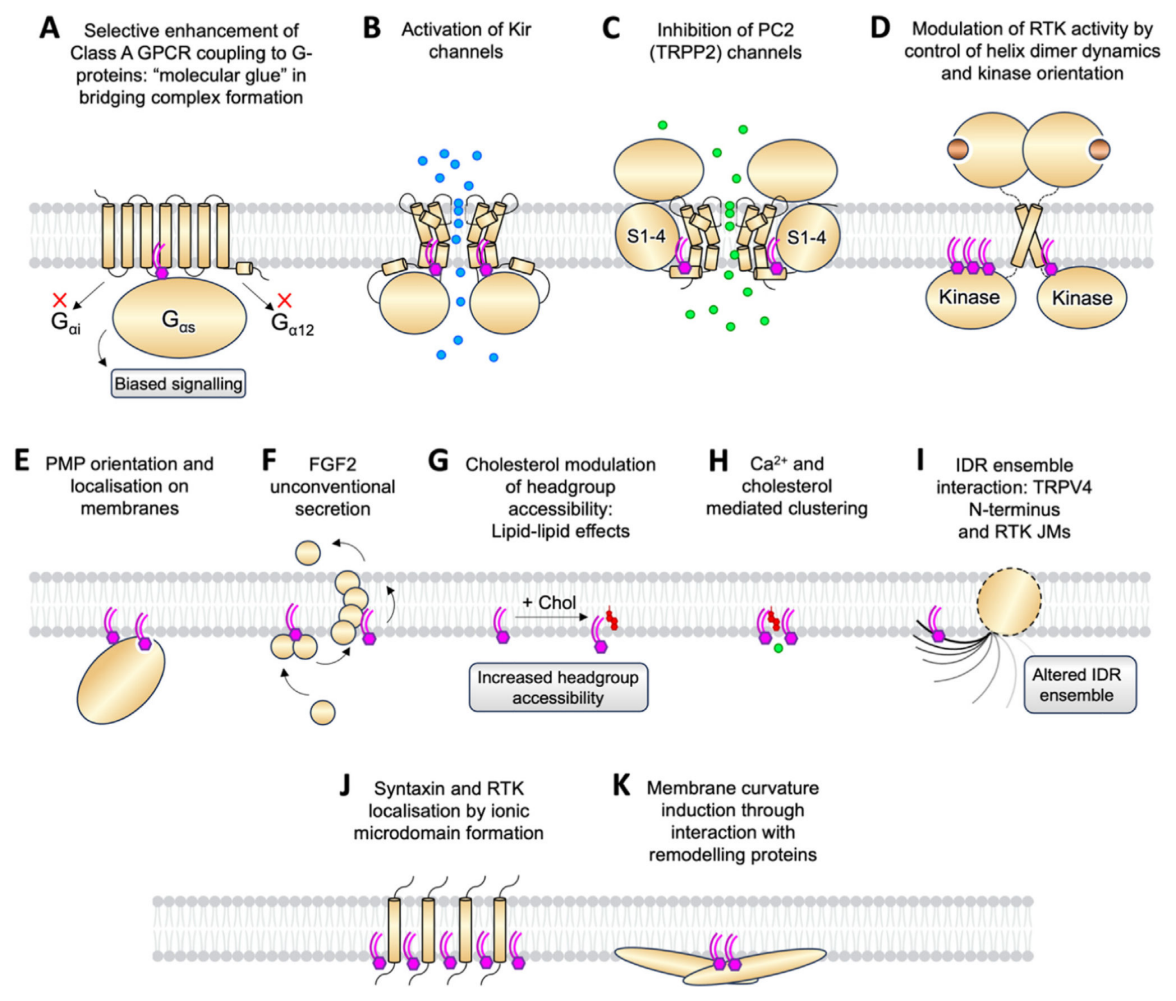
Cellular roles of phosphoinositide lipids studied by simulations. (A) Combined CG MD simulation and nMS based discovery of GPCR binding and functional modulation of G-protein coupling by PI(4,5)P_2_.^21^ (B) Multiscale simulation based identification and characterisation of PI(4,5)P_2_ binding sites on Kir channels.^84,199,206,214^ (C) CG MD simulation identification of a phosphoinositide binding site on PC2 with functional consequence.^181,182^ (D) Multiscale simulation of PI(4,5)P_2_ control of RTK kinase domain orientation, and TM-JM dynamics.^189,293,295,303^ (E) CG, all-atom, and HMMM simulation of PI(3)P, PI(4)P, PI(4,5)P_2,_ and PI(3,4,5)P_3,_ interaction with many PMPs: see section 2.3. (F) All-atom simulations of PI(4,5)P_2_ in FGF2 secretion.^99,307^ (G) All-atom simulations of cholesterol – PI(4,5)P_2_ interplay.^99^ (H) Multiscale simulations of phosphoinositide clustering.^331,332,85,99,334^ (I) Multiscale simulation of PI(4,5)P_2_ interaction with IDRs within receptors^189^ and channels.^191^ (J) Combined CG MD simulation and microscopy of phosphoinositide microdomain formation.^38,189,284^ (K) Multiscale and ultra-CG simulations of PI(4,5)P_2_ interaction with remodelling proteins.^267,335,336,338,339^

Simulations have been applied in a variety of ways to address the molecular underpinnings of how phosphoinositides influence membrane proteins (Figure 10). By comparison to experiment it is evident they are capable of accurately identifying phosphoinositide binding sites. This was the case for Class A GPCRs^21^ and Kir channels,^84^ as confirmed by later X-ray^17^ and cryo-EM^140^ structures. Simulations may also be used synergistically to interpret ambiguous experimental lipid density in structures.^23^ Wang et al., thus used CG-simulations to identify a general phosphoinositide binding site on the PC2 ion channel, in agreement with their cryo-EM structures.^181^ This general phosphoinositide site was later refined to a possible PI(4)P site in a subsequent biochemical and cell-based functional study.^182^ Characterisation of the energetics of phosphoinositide interaction may be achieved using advanced simulation techniques such as FEP-derived ΔΔGs for lipid binding,^199^ and PMF-derived free energy landscapes for PMP association with membranes from solution.^248^ Analysis of the interaction patterns in simulations of different conformational states can show in atomic detail how functional effects are conferred by structure, for instance in PI(4,5)P_2_ stabilisation of the open state of Kir2.2.^199,214^ Function may also be prospectively predicted from the structure and dynamics seen in a simulation, for instance in simulation-based prediction that PI(4,5)P_2_ lipids act as a molecular glue to bridge A_2A_R – miniG interactions to enhance G-protein coupling, in agreement with nMS andfluorescence-based functional assays.^21^

Increases in computational power^110^ and structure availability^18,143^ increasingly allow assessment of phosphoinositide interactions at scale across large groups of membrane proteins, for instance RTKs,^189^ GPCRs,^149^ and PH domains,^249^ facilitating assessments of conservation. Expansion of parameter space for phosphoinositides,^83^ and membrane lipids generally^69,86,95,342^ now allow routine simulation in complex lipid mixtures, which have revealed new aspects of phosphoinositide biology deriving from lipid-lipid interplay.^99,199^ The vast majority of simulation studies to date have focussed on PI(4,5)P_2_, which likely derives from its localisation in the plasma membrane, where membrane protein research has tended to focus. It will be especially welcome to see phosphoinositide research expand further into subcellular membranes containing other phosphoinositide species.

Existing limitations of phosphoinositide simulations, such as fixed charge force fields, and challenges in distinguishing closely related isomers e.g. PI(3,5)P_2_ and PI(4,5)P_2_ in CG models^181^ have also been seen. Continued developments in multiscale modelling,^74^ and constant pH simulations^343,344^ will be especially valuable in this area. The CG MARTINI model has shown exceptional utility in *de novo* prediction of phosphoinositide binding sites,^64,72^ (converged) free energy calculations,^108,199,345^ and modelling of large membrane phenomena.^38,166^ At the same time, we would caution the budding explorer of phosphoinositide biology against the risk of overinterpretation of CG models. For instance, in capturing the detailed pattern of phosphoinositide interactions and ensemble of poses within a given binding site, certain ion-induced effects,^85^ and differentiation between closely related isomers. Moreover, high-quality (ideally not detergent or chemical probe based) experimental measures of specific lipid interaction energies with membrane proteins are scarce, which makes direct comparison to CG interaction energies challenging.^108^ All-atom simulations (includingflavours such as HMMM^92,245^) have tended to excel in areas such as refinement of detailed interactions with known binding sites,^206,237^ specific ion interactions,^85,329,332^ and phosphoinositide-induced membrane protein conformational change.^214,242^ Ultra-CG models^346^ have also shown utility in modelling of large-scale phenomena such as membrane protrusion formation by I-BAR domains.^335^

In considering the present body of phosphoinositide simulations it is evident that they interact with a huge range of membrane proteins. Unsurprisingly these interactions nearly always feature basic residues on the protein. In the case of integral membrane proteins, these residues tend to be positioned slightly beyond the membrane surface, and are thus optimally positioned to interact with the 3′, 4′, and 5′ phosphoryl groups of phosphoinositides. Such positions are often inaccessible to other phospholipids with smaller headgroups and this is one way specificity is conferred. Given the body of simulation work on phosphoinositides, it should now be possible to derive more detailed heuristics on what characterises a phosphoinositide binding site. Can a suite of pharmacophore and QSAR models for phosphoinositides be developed? Can machine learning technologies be trained on the abundance of simulation and structural data on phosphoinositide interactions with membrane proteins? Leveraging simulation data in this way is a key challenge for computational biochemists. At present simulation data is generally stored individually within the filesystems of research groups, which severely circumscribes large scale meta-analysis of this nature. Upcoming initiatives such as the Molecular Dynamics Data Bank (MDDB)^347^ (see https://mddbr.eu/) and MDverse^104^ will be welcome advancements and should enable new insights into phosphoinositide biology. Such analysis would be especially welcome from a proof-of-concept therapeutic perspective, as a principal challenge to leveraging lipid binding sites on membrane proteins for structure-based drug design^348,349^ is likely to be selectivity.

The advent of AI models for biomolecular structure prediction is expected to further expand the range of questions which can be addressed by physics-based simulations. The widespread availability of increasingly accurate structure predictions of proteins and their conformations will provide new entry points from which to initiate dynamical simulations. This effect will be felt particularly for simulations of membrane proteins, where experimental structure determination remains challenging compared to soluble proteins.^350^ Recent developments in AI models which predict small molecules in complex with target proteins^54,56,57^ are anticipated to be especially valuable in rapid prediction of phosphoinositide binding sites on membrane proteins, and will serve to provide additional points from which to launch detailed simulation and experimental studies. We also note the evolving ability of cryo-EM to capture time-resolved conformational heterogeneity,^351,352^ offering the intriguing possibility for further simulation – experimental synergy in a temporal dimension.

Simulations have grown to occupy a unique place in the toolkit available to the interested connoisseur of phosphoinositide biology. Their synergistic application hand in glove with rigorous structural and functional experiment is likely to continue to reveal new facets of the molecular underpinnings of the cellular effects of phosphoinositides.

## Summary

Hedger and Yen review developments in simulations of phosphoinositides and membrane proteins.

## Abbreviations

MD: molecular dynamics
CG: coarse-grained
cryo-EM: cryogenic electron microscopy
nMS: native mass spectrometry
AI: artificial intelligence
ML: machine learning
NMR: nuclear magnetic resonance
ESR: electron spin resonance
EM: electron microscopy
PC: phosphatidylcholine
PG: phosphatidylglycerol
PS: phosphatidylserine
PE: phosphatidylethanolamine
PI: phosphatidylinositol
PI(4)P: phosphatidylinositol-4-phos phate
PI(4,5)P_2_: phosphatidylinositol-4,5-bisphosphate
PI(3,4,5)P_3_: phosphatidylinositol-3,4,5-trisphosphate
FEP: free energy perturbation
PMF: potential of mean force
GPCR: G-protein-coupled receptor
NTSR1: neurotensin receptor 1
A_2A_R: Adenosine_2A_ receptor
RTK: receptor tyrosine kinase
JM: juxtamembrane
TRP: transient receptor potential
PC2: polycystin-2
Kir: inward rectifying potassium ion channel
PMP: peripheral membrane protein
